# P16 The national IV to Oral Antibiotic Switch (IVOS) Commissioning for Quality and Innovation (CQUIN) scheme; exploration of non-submission through semi-structured interviews

**DOI:** 10.1093/jacamr/dlaf230.023

**Published:** 2025-12-04

**Authors:** Rachel Berry, Bee Ng Yean, Kieran Hand, Monsey McLeod, Hugh Attwood, Diane Ashiru-Oredope

**Affiliations:** UK Health Security Agency, London, UK; UK Health Security Agency, London, UK; NHS England, London, UK; NHS England, London, UK; NHS England, London, UK; UK Health Security Agency, London, UK; University of Nottingham, Nottingham, UK

## Abstract

**Background:**

The national IV to oral switch (IVOS) criteria for adults prescribed antibiotics were published in November 2022. This guidance encourages timely switching from IV to oral antibiotics when clinically appropriate, aiming to improve antimicrobial stewardship (AMS), reduce hospital stays and lower costs. In 2023–24 a national Commissioning for Quality and Innovation (CQUIN) scheme for IVOS was introduced for acute hospital Trusts in England to incentivize the implementation of this guidance; setting a target of 40% (or fewer) patients still receiving IV antibiotics past the point at which they meet switching criteria as demonstrated by patient-level audit data submission.

**Objectives:**

As part of evaluating the national AMS intervention, the aim of this study was to explore barriers to submission for the national IVOS CQUIN and facilitators and barriers to appropriate IVOS or reduction in IV antibiotic (IVAB) use in those Trusts that did not submit CQUIN data but saw reductions in overall IVAB consumption per admission.

**Methods:**

Lead AMS staff from all trusts that did not submit CQUIN data but had a reduction in IVAB consumption per admission in 2023–24 compared to 2022–23 were contacted via email to invite to take part in a semi-structured interview; consent was received in writing in advance. The interviews were conducted over Microsoft Teams^®^ and transcriptions were anonymized for data extraction. Transcripts were coded using NVivo^®^ v15. This exploratory evaluation study was approved by the UKHSA Research Ethics and Governance Group (NR0408).

**Results:**

Of the ten trusts identified as eligible, representatives from nine trusts consented to the interview. Analysis of the interviews showed that a third of Trusts did not submit CQUIN data as they were exempt or felt it was not appropriate due to being a specialist Trust. The remaining six did not submit due to lack of capacity within the pharmacy workforce or the wider stewardship team. Regarding specific work to increase IVOS or reduce IVAB use, one Trust had attempted to implement changes but had not been successful within the time frame, one believed that there was a data error and three trusts did not do any specific work. Key themes of facilitators from the remaining four Trusts included implementation of specific IVOS projects supported by a key person with dedicated time, a focus on empowerment and education of non-medical staff to encourage review of IV antibiotics and use of national evidence-base or guidance to support local initiatives and policies around IVOS or reducing IVAB use, although few highlighted the national IVOS checklist or CQUIN within this.

**Conclusions:**

This qualitative study identified that the key reason for Trusts not submitting data for the national IVOS CQUIN 23/24 was due to lack of workforce capacity. Some Trusts did not carry out any specific activities, hence were unsure why the IV consumption per admission in their trust had reduced. However, analysis of key themes of facilitators identified in those Trusts that did do specific work could offer opportunities for scaling up at a national level to improve timely IVOS.

